# Intake of fruits and vegetables (FAVs) on cognitive functions among adolescents and young adults: a scoping review

**DOI:** 10.1017/jns.2025.10012

**Published:** 2025-11-20

**Authors:** Joyce Sangeetha Soans, Judith Angelitta Noronha, Suneel C. Mundkur, Baby S. Nayak, Meenakshi Garg, Sonia R.B. D’Souza, Roshan David Jathanna, Namratha Pai Kotebagilu, Revathi P. Shenoy, Ravishankar Nagaraja, Pratibha Kamath

**Affiliations:** 1 Department of OBG Nursing, Manipal College of Nursing, Manipal Academy of Higher Education, Manipal, Karnataka, India; 2 Department of Child Health Nursing, Manipal College of Nursing, Manipal Academy of Higher Education, Manipal, Karnataka, India; 3 Department of Paediatrics, Kasturba Medical College, Manipal Academy of Higher Education, Manipal, Karnataka, India; 4 School of Computer Engineering, Manipal Institute of Technology, Manipal Academy of Higher Education, Manipal, Karnataka, India; 5 Department of Biochemistry, Kasturba Medical College, Manipal Academy of Higher Education, Manipal, Karnataka, India; 6 Department of Biostatistics, Vallabhbhai Patel Chest Institute, University of Delhi, Delhi, India; 7 Department of Dietetics and Applied Nutrition, Welcomegroup Graduate School of Hotel Administration, Manipal Academy of Higher Education, Manipal, Karnataka, India

**Keywords:** Adolescents, Cognitive functions, Fruits, Scoping review, Vegetables, Young adults, FAV, Fruits and Vegetables, CF, Cognitive function, CVD, Cardiovascular disease, WHO, World Health Organization, MCI, Mild Cognitive Impairment, AD, Alzheimer’s disease, WOS, Web of Science

## Abstract

This scoping review provides an overview of the impact of fruit and vegetable (FAV) consumption on cognitive function in adolescents and young adults between January 2014 and February 2024. A comprehensive search across six databases, CINAHL, PubMed-MEDLINE, ProQuest, Web of Science, Scopus, and Embase, identified 5,181 articles, of which six met the inclusion criteria after deduplication and screening. This scoping review focused on individuals aged 11–35 years in schools, colleges, universities, and communities. Following a descriptive and narrative synthesis of the data, tables and figures were used to present the findings. Across the six included studies, most consistently demonstrated a positive association between higher fruit and vegetable (FAV) intake and improved cognitive performance among adolescents and young adults. This association was evident in both cross-sectional and longitudinal studies, with stronger effects observed for whole fruits and vegetables high in fibre and polyphenols. Cognitive domains positively impacted included psychomotor speed, memory, attention, and mood. However, findings varied by type of food and cognitive domain; while whole FAVs were generally beneficial, results for fruit juice were mixed—some studies showed acute benefits. Differences in study designs, dietary assessment tools, and cognitive measures contributed to variability. Despite these inconsistencies, the overall trend supports a beneficial role of FAV consumption in promoting cognitive health during adolescence and early adulthood. This review demonstrates that increased fruit and vegetable consumption is consistently linked to improved cognitive function in adolescents and young adults. However, further research is needed to establish its long-term effects on cognitive ageing and disease prevention

## Introduction

Cognition encompasses a variety of mental processes, including learning, reasoning, memory, attention, language creation, comprehension, and problem-solving.^(1)^ Cognitive decline is related to age and is independently associated with mortality.^(2)^ Faster cognitive decline is linked to an increased risk of certain diseases, such as diabetes, hypertension, and cardiovascular vascular disease (CVD),^(3)^ In turn, this puts individuals at risk of chronic and lifetime illnesses. In most cases, a slow process of brain atrophy affects cognitive function, usually around the age of 30.^(4)^

Throughout the life course, dietary intake plays a significant role in cognitive outcomes. It is observed that the majority of adolescents do not consume a sufficient amount of fruits (45%) or vegetables (30%).^(5,6)^ During adolescence, it’s essential to maintain good health because the habits developed during this time can continue into adulthood.^(7)^ Introducing a high-quality diet to young adolescents in early life may have a positive effect on their health. Studies have shown that children younger than three years who ate a healthy diet had higher academic scores.^(8)^ Nutrition quality early in life may play a significant role in academic performance in the future.^(9–11)^ Age-related mild cognitive impairment (MCI) can also be avoided or delayed with the support of healthy eating habits.^(12,13)^ An increasing amount of evidence indicates that a diet high in plants is crucial for preventing cognitive decline since fruits and vegetables are rich in antioxidants, which frequently have a protective effect on sustaining cognitive performance.^(14)^

Fruits and vegetables, which have been shown to improve cognitive functions, include green leafy vegetables, apples, almonds, berries, grapes, cruciferous vegetables, spices, and olive oil.^(14)^ Beetroot juice and berries have been found to enhance brain signalling.^(15,16)^ Berry fruits such as blueberries and blackcurrant berries are rich in polyphenols and offer numerous health benefits, such as reduced inflammation, cardiovascular risk, anticancer effects, and protection against neurogenerative disorders. Among them, the one with the highest polyphenol content was purple grape juice, which contains 65% Concord grape.^(17)^ Certain dietary ingredients, particularly polyphenols, have been linked to cognitive function and mental health. Many foods and beverages contain polyphenols, including vegetables, fruits, herbal teas, chocolate, green tea coffee, alcoholic beverages and red wine.^(18)^ However, some concerns still need to be raised.

First, the current available information is mostly based on research conducted in Western nations. More data is needed on the connections between adolescents, adult cognitive performance, and the consumption of FAV’s. Multiple studies have indicated that higher intake of FAV’s by individuals with high levels of physical activity can lower the risk of cognitive decline. These findings suggest that physical activity and consumption of FAV may have combined effects in reducing cognitive decline.^(19)^ Urbanisation and accelerated economic development have drastically changed dietary habits, shifting food demand.^(20)^ For instance, a recent report highlighted that daily fruit intake is particularly low in South and East Asia, where middle-aged adults did not meet World Health Organization FAV recommendations. The NHFS study conducted in 2005–2006 and again in 2019–2021 revealed a decline in total protein consumption and dark green leafy vegetables, while fruit consumption remains low. Despite half of the Indians regularly eating foods from animals, they do not consume protein in legumes or animal products.^(21)^

In contrast, those from Western nations typically eat a balanced diet of fruits and vegetables in roughly equal proportions. More than two-thirds (67.3%) of adults in the United States aged over 20 years consume fruit daily. Of these, 47.5% consumed citrus fruits, melons, berries, and other whole fruits, and 30.8% consumed 100% of the fruit juice. The evidence provided indicates that the habits underlying the intake of FAV’s among adults in non-Western cultures may vary and require additional examination.^(22,23)^ Furthermore, despite the potential for distinct protective effects,^(24)^ the literature frequently discusses the overall effect of the consumption of both FAVs. For example, consuming enough vegetables, mainly those high in fibre, and fruits may help to sustain cognitive function by promoting long-term weight control and decreasing the chances of developing conditions such as metabolic syndrome and diabetes.^(25,26)^ The presence of inorganic nitrates found in leafy green vegetables might enhance cognition by safeguarding cardiovascular health.^(27)^ Hence, the current review examines the association between eating fruits and vegetables and cognitive functioning in adolescents and young adults.

This review is highly important for low- and middle-income and developing countries where the proportion of older adults is increasing, along with the prevalence of Alzheimer’s disease and mild cognitive impairment. These findings emphasise the importance of eating healthy in preserving cognitive function among younger individuals.^(13)^ By promoting a diet rich in fruits and vegetables, societies can potentially reduce the future burden of MCI and AD on families and healthcare systems.

## Methods and materials

The study adhered to the recommendations made by Arksey and O’Malley for scoping reviews. After screening the data from the available databases using these guidelines, we presented the results in compliance with the PRISMA extension for the scoping review.

### The eligibility criteria

Studies were chosen based on the following eligibility criteria.

#### Participants

Adolescence and young adults ranging from 11 to 35 years of age were included in the study.

#### Setting

Schools, colleges, universities, and communities.

#### Outcomes

Eligible studies reported on dietary patterns, fruit and vegetable intake, fruit juice intake and interventions to improve cognitive function.

#### Study design

Randomised control trials, longitudinal and cohort studies.

#### Time frame

From January 2014 to February 2024. Studies published in various databases, grey literature, and manual research were included.

#### Language

Studies conducted exclusively in English were considered.

#### Exclusion criteria

We excluded articles that did not meet the inclusion criteria and did not examine the association between dietary intake of FAVs and cognitive function. Additionally, we excluded articles that included systematic reviews, meta-analyses, conference articles, reports, or reviews. We did not include any studies that were completed before 2014. Studies completed before January 1, 2014, or in a language other than English were excluded.

### Information sources and search strategy

Our current literature review was conducted with the assistance of a research librarian to locate the most current literature on a topic that has recently gained significant attention. We searched six online databases: CINAHL, PubMed, EMBASE, SCOPUS, WOS, and ProQuest. Grey literature searches and manual searches were also conducted (the Government of India database and Shodhaganga). Studies published between January 1, 2014, and February 29, 2024, included in the search. SJS and JAN collaborated to devise the search strategy, which was formulated based on the study’s primary research question. This strategy was then cross-checked with relevant keywords and databases pertinent to the study, as identified by SRBD, SM, and a librarian. Additionally, MESH terms and free text of keywords were used as required.

Keywords included ‘Adolescent’, Adolescence’, ‘Young Adult,’ ‘Teen’ Youth OR ‘Young person’ OR ‘Youngsters’ and ‘eating’, ‘feeding behaviour’ and ‘fruits’, ‘vegetables’ and ‘cognitive functions’, ‘executive functions’, and ‘cognitive impairment’. We used Boolean operators appropriately. The search strategy was initially created for PubMed before developing a strategy for the other databases.

### Selection of the relevant studies

As a result of deduplication, 5181 articles were found during the initial search. Two independent reviewers screened the articles. Three-stage screening and data extraction procedures (including the title, abstract, and full text) were carried out independently by two authors (SJS and JAN), who adhered to the eligibility requirements as per the data extraction form. Eighteen articles were chosen for full-text review after the initial screening, after which the findings were compared and discussed. Six articles were determined to fit the inclusion requirements after a thorough assessment. The third author resolved any differences (MG). In conversation with the lead reviewers and the advisory group members, all discrepancies discovered throughout the screening, data extraction, and analysis processes were resolved. (SRBD, SM, RS, and RV). The results were managed and screened using Rayyan software.

### Data extraction/charting

We extracted data on population (i.e. adolescents and young adults), study location, sample size, study design, focus on the objective of the study, standardised tools used for dietary intake, standardised tools used to assess the cognitive functions, and outcomes, i.e. dietary outcomes. The data were extracted in Microsoft Excel 2013 (SJS & JAN). The first author (SJS) extracted the data, which were subsequently verified by a second author (JAN). Disagreements between the two authors were settled through discussion.

### Data analysis and synthesis

Microsoft Excel 2013 was used for data analysis. The study examined the association between fruit and vegetable (FAV) intake and cognitive function using descriptive statistical measures, including frequencies, percentages, confidence intervals (CI), and p-values. The data analysis focused on assessing dietary intake patterns, cognitive function measures, and their associations. A descriptive approach was utilised to summarise findings, with results presented in tables. Data extracted for each table were categorised based on study methodology, dietary intake assessment tools, cognitive function assessment tools, and outcome measures. The synthesis process involved grouping studies according to study design, participant characteristics, and dietary exposure variables to identify patterns in FAV intake and cognitive outcomes. (Refer to Tables 1 and 2)

**Table 1.** Characteristics of the included studies

Author & year	Study location	Population /age group	Study design	Study setting	Sample size	Data collection tools Dietary assessment tools	Data collection tools Cognitive assessment tools
Nyaradi et al. 2014	Western Australia	Adolescents 14–17 years	Cohort Prospective	King Edward memorial Hospital, western Australia	2,868 (gender not mentioned)	Food frequency questionnaire	CogState (CogState Ltd, Melbourne, Australia) computerised cognitive battery, Detection task. The main cognitive domains measured are as follows: psychomotor functioning (DET), visual attention(IDN), memory and attention (OCL), visual learning and mem-ory (CPAL), and memory and executive function (GML and GMR)
Mao Xuanxia et al.	United States of America	18–30 years	Longitudinal cohort	4 study centres in United States	3231 women – 1,644, men – 1,587	A CARDIA Diet history questionnaire	Cognitive test – Rey Auditory Verbal Learning Test (RAVLT), Digit Symbol Substitution Test (DSST), Stroop test
Chen Jing et al. 2023	Finland	3–18 years of age. (Mean age 11.2 years)	A prospective cohort	Cardiovascular Risk in Young Finns Study (YFS), five university cities with medical schools and their rural surroundings	3596 children (3–18 years) boys – 1,819 girls – 1,777	Six dietary patterns were derived from 48-h food recall or food frequency questionnaires.	Cognitive function outcomes assessed included episodic memory and associative learning, short-term working memory and problem solving, reaction and movement time, and visual processing and sustained attention.
Khalid Sundus, et al. 2017	United kingdom	18–21 years young adults	Double-blind, placebo-controlled, crossover study.	University	21 (19 females and 2 males)	I: The flavonoid-rich wild blueberry (WBB) drink contained 253 mg anthocyanins and was prepared by mixing 30 g of freeze-dried WBB with 30 mL of low-flavonoid Rocks Orange Squash and 220 mL of water C: The placebo drink was matched to the WBB drink for vitamin C (4 mg), sugars (8.90 g fructose, 7.99 g glucose), 30 mL Rocks Orange Squash and 220 mL of water	The Positive and Negative Affect Schedule-NOW (PANAS-NOW) assessed current mood. (10 posts and 10 negative mood states). Positive and Negative Affect Schedule – Now version
Ramsay Haskell C.F, 2017.	United Kingdom	18–35 years	Randomised placebo-controlled, double-blind, counter balanced -cross over design	University department of psychology	20 participants (7 males, 13 females)	I:. The active treatment consisted of 200 ml Welch’s™ purple grape juice (based upon single serving guidelines at the time of the study) plus 30 ml of Schweppes™ blackcurrant favour cordial (containing 14 kcal and 3.1 g sugar per 100 ml). C: Placebo consisted of 200 ml Welch’s™ white grape juice plus 10 ml blackcurrant favour cordial and 20 ml cold water. Schweppes™ blackcurrant favour cordial was added at different volumes to the active and placebo drinks to ensure matched sugar content, this also served to mask the favour of the drinks	All cognitive and mood measures were administered using the Computerised Mental Performance Assessment System. Memory task were assessed (immediate and delayed word recall, numeric working memory, word recognition, picture recognition) or an attention task (simple reaction time, choice reaction time, digit vigilance). Subjective mood- Bond Lader mood scales
Lamport J Daniel et al. 2017	United Kingdom	Healthy young adults 18–30	Single-blind, randomised, cross-over design	University of Reading	24 participants (4 men and 20 women)	I: high-flavanone (HF; 70·5 mg) drink. C: an energy-, and vitamin C matched, zero-flavanone control	Cognitive behavioural testing and ASL measurements were performed. Cogntive Battery Test -Word Recall (immediate), Logical Memory (immediate recall), Sequence Learning Task, Digit Symbol Substitution Test (DSST), Stroop Test, Letter Memory Test, Go-NoGo Task, Spatial Delayed Recall, Word Recall (delayed) and Logical Memory (delayed).

DET: Detection Task – measures **psychomotor functioning** (reaction time; lower score = better performance), IDN: Identification Task – measures **visual attention** (reaction time; lower score = better performance), OCL: One Card Learning Task – measures **memory and attention** (correct responses; higher score = better performance), CPAL: Continuous Paired Association Learning Task – measures **visual learning and memory** (total errors; lower score = better performance)GML: Groton Maze Learning Test – measures **executive function** (total errors; lower score = better performance)GMR: Groton Maze Learning Test – Delayed Recall – measures **memory recall** (total errors; lower score = better performance).

RAVLT: Rey Auditory Verbal Learning Test (verbal memory), DSST: Digit Symbol Substitution Test (executive function and psychomotor speed).

PANAS-NOW: Positive and Negative Affect Schedule – Now version.

**Table 2.** Association of fruits and vegetables on cognitive functions

Author & year	Objectives	Duration	Follow up	Findings
Nyaradi Anett et al. 2014	To investigate prospective associations between dietary patterns and cognitive performance during adolescence	3 years 2006–2009	Baseline line at 14 and 3 years later.	Within the dietary patterns, high intake of fried potato, crisps and red meat had negative associations, while Increased fruit and leafy green vegetable intake had positive associations with some aspects of cognitive performance. Higher dietary pattern at the age of 14 years is associated with diminished cognitive performance 3 years later at the age of 17.
Mao Xuanxia et al. 2019	To examine the long-term association between VF intakes, including VF subgroups, in young adulthood and cognitive function in midlife	25 years	Baseline, 2,5,7,10,15,20,25, and 30 years	Intake of whole vegetables was significantly associated with better cognitive performance after adjustment for potential confounders in all 3 cognitive tests. (quintile 5 compared with quintile 1RAVLT, 95% CI: 0.01, 0.64; p = 0.08; DSST, 95% CI: 0.93, 4.75; p < 0.01; Stroop test, 95% CI: −4.24, −1.50; p-< 0.01]. Similarly, the intake of fruits, except fruit juices, was significantly related to better cognitive performance (quintile 5 compared with quintile 1—DSST, 95% CI: 0.70, 4.12; p = 0.03). This study supports the long-term benefits of VF consumption on cognitive performance, except those VF with relatively low fibre content such as potatoes and fruit juices.
Chen Jing et al. 2023	To examine the association of youth, adulthood, and long-term dietary patterns from youth to adulthood with cognitive function in midlife.	1980–2011	4 follow-ups (1980 baseline, 1986, 2001, 2007, 2011)	Both youth and long-term vegetable and dairy products and healthy patterns were positively associated with episodic memory and associative learning scores (p < 0.05 for all). Higher adherence to healthy and vegetable and dairy product patterns was associated with better cognitive function. The findings, if causative, highlight the importance of maintaining a healthy dietary pattern from early life to adulthood in an attempt to promote cognitive health.
Khalid Sundus, et al. 2017	Benefits of flavonoid on executive function.	1 years	No follow up	There was a significant increase in PA after consuming the WBB drink (pre: M = 22.76, SD = 9.01 and post: M = 29.48, SD = 8.60; t(20) = −4.68, p < 0.001). There was no change in PA after consuming the placebo drink (pre: M = 24.0, SD = 9.59 and post: M = 25.24, SD = 8.50; t(20) = −0.73, p = 0.48. thus, blueberry intervention increased positive affect but had no effect on the negative affect.
Ramsay Haskell C.F	Benefits of purple juice on cognitive functions	1 year	No follow up	Purple grape juice significantly improved reaction time on a composite attention measure (p = 0.047) and increased calm ratings (p = 0.046) compared to placebo. Order effects also indicated an enduring positive effect on pre-dose memory reaction time (p = 0.018) and post-dose calm ratings (p = 0.019) when the purple grape was consumed first. Purple juice enhances cognition and mood in young adults.
Lamport J Daniel et al. 2017	To investigate whether consumption of flavanone-rich juice is associated with acute cognitive benefits and increased regional CBF in healthy, young adults	One year	Baseline and after two days.	The post hoc t-tests revealed that consumption of the HF drink resulted in a significant improvement in DSST performance at two relative to baseline (t=3·84, p < 0·01). No significant improvement in performance was observed following the CT drink (t=0·05, p = 0·96). Baseline DSST performance did not differ between the CT and HF drinks (t=0·02, p = 0·98). No significant interactions or main effects were observed for all other cognitive tests. These results demonstrate that consuming flavanone-rich citrus juice in quantities commonly consumed can acutely enhance blood flow to the brain in healthy young adults. However, further studies are required to establish a causal link between increased CBF and improved behavioural outcomes following citrus juice ingestion.

FAV: Fruits and Vegetables, CF: Cognitive Function, RCT: Randomised Controlled Trial, DET: Detection Task (CogState domain: psychomotor function), IDN: Identification Task (CogState domain: visual attention), OCL: One Card Learning Task (CogState domain: memory and attention), CPAL: Continuous Paired Association Learning Task (CogState domain: visual learning and memory), GML: Groton Maze Learning Test (CogState domain: executive function), GMR: Groton Maze Recall Test (CogState domain: memory recall), RAVLT: Rey Auditory Verbal Learning Test (verbal memory), DSST: Digit Symbol Substitution Test (executive function and psychomotor speed), CBF: Cerebral Blood Flow, CT: Control HF High Flavanone.

## Results

**Fig. 1.**
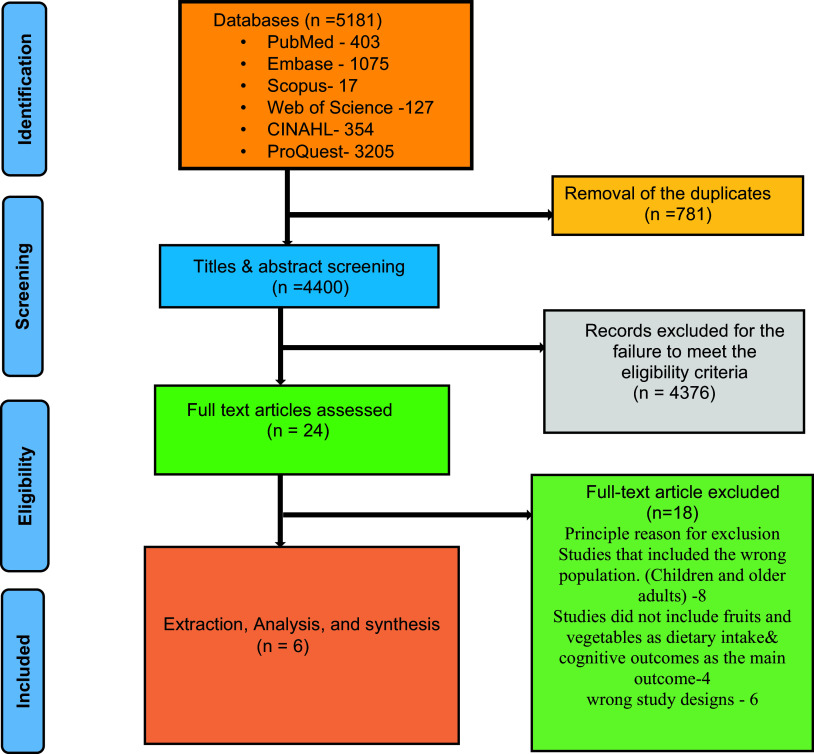
A PRISMA flowchart of the study selection process.

A total of 5181 records were subjected to title screening after 718 duplicate records were removed, and 4745 of those records were included. We conducted an abstract screening process to complete a full-text screening of 6 studies because they met the inclusion criteria. A flow diagram of the PRISMA study selection process is shown in Figure 1.

Among the six studies,^(17,28–32)^ three studies^(17,29,30)^ were conducted in the United Kingdom, and the other studies were conducted in Australia,^(28)^ the USA,^(31)^ and Finland.^(32)^ Table 1 summarises the study design, study location, population, sample size, and data collection tools used for both dietary intake and cognitive function. The studies comprised two cohort prospective studies, one longitudinal prospective study, and three randomised controlled trials, with a total sample size of 9,578 participants aged 11–35. The longitudinal studies^(17,29,30)^ explored the relationship between childhood and adulthood dietary habits and midlife cognitive function patterns. Another study^(31)^ examined the association between dietary patterns and cognitive performance during adolescence, and each of the included studies included additional covariates in their analyses. The data included demographic information, Western dietary patterns, maternal education, family history, income, gender, and family functioning. Additionally, it encompassed total energy intake, vitamin intake (A, B, and C), and potassium intake. The US study^(31)^ analysed the intake of FAV and information on cognitive function among young adults and on cognitive function in midlife. Three other studies^(17,29,30)^ reported that fruit juice intake enhances cognition and mood. Most of the additional research included randomised clinical trials of dietary intake, where cognitive function was not the primary outcome of the intervention being evaluated.

### Identification and definitions of dietary intake of fruits and vegetables

All six studies^(17,28–32)^ clearly defined the identification of adolescents and young adults. The population of teenagers aged 14–17 years who participated in one study was drawn from the Western Australian Pregnancy Cohort.^(28)^ In another study^(32)^ participants aged 18–30 were recruited from the national register of five Finnish universities with medical schools and rural areas. In the United States,^(31)^ study adolescents aged 18–30 years were recruited from four study centres, ensuring an even distribution by sex, race, and educational level within each centre. A study conducted in the United Kingdom^(30)^ recruited participants aged 18–35 through community advertising via emails, posters, and leaflets. Additionally,^(29)^ undergraduate students aged 18–25 from the University of Reading participated in another study. Furthermore, all participants in the^(17)^ Newcastle study aged 18–30 years, who were not pregnant or planning for pregnancy; lactating; had no medical, neurological, vascular, or psychiatric illnesses; had no history of drug, alcohol, caffeine intake, or smoking abuse; were not on medications or dietary supplements or any history of allergies.

### Measures of dietary intake of FAV

In all six studies,^(17,28–32)^ the Food Frequency Questionnaire (FFQ) was used as the primary measure of Dietary Intake (DI). For example, the semiquantitative FFQ and 48-hour dietary recall methods, as well as the interview method, were used in two studies.^(30,32)^ In one study,^(31)^ the Coronary Artery Risk Development in Young Adults (CARDIA) Diet History Questionnaire was used to categorise the reported foods into 166 subgroups within nine major food groups. One study^(32)^ used a 48-hour dietary recall method in which trained dietitians asked participants to write down the type and amount of food they ate the two days before the interview. After a period of time, the participants were also asked to complete a well-researched 131-item food and fitness questionnaire.

### Measures of dietary intake of fruit juices

In all three studies,^(17,29,30)^ the intervention group received juices, i.e., purple grape juice,^(17)^ flavonoids^(30)^ and blueberries,^(29)^ whereas a placebo (drinking that matched the content of the intervention group) was provided to the non-intervention group. In all three studies, the drinks were provided in opaque cups with straws to blind the participants.

Purple grape juice plus additional tastes were given to the treatment group in one trial,^(15)^ whereas the control group received a placebo. The favour cordial was added at different volume levels to ensure a similar sugar content between the active and placebo drinks.

In another study,^(30)^ the 500-ml HF drink was a commercially available 100% juice, which naturally contained flavonoids, while the other group received the same commercially concentrated cordial product derived from mineral water containing no flavonoids. In another study,^(29)^ flavonoid-rich wild blueberries (WBB) were compared to a placebo drink.

### Cognitive function measures

All six studies,^(17,28–32)^ used different standardised scales to assess cognitive functions. One study measured cognitive performance using the CogState.^(28)^ The test was administered in a variety of cultural contexts with only a minimal demand for language proficiency. Since a deck of cards measures cognitive function, this computerised testing method proved particularly pertinent for children and adolescents. The primary results were derived from seven different CogStates: detection, identification, one-card learning, continuous paired association learning, the Groton Maze Learning Test, and the Groton Maze Learning Test – delayed recall. Additionally, six primary cognitive functions were assessed: psychomotor functioning, memory and attention, visual learning and memory, visual attention, and executive function. The three tests used by the authors of one study^(31)^ to assess cognitive function were the Stroop test, which measures inhibitory control; the digit symbol substitution test (DSST), which measures sustained attention, psychomotor speed, and sustained memory, and the Rey Auditory Verbal Learning Test (RAVLT), which measures verbal learning and memory. Another study also incorporated a computerised cognitive testing battery, which assesses spatial working memory, paired association learning tests, reaction time tests, and quick visual information tests for cognitive evaluation.^(32)^ In randomised controlled trials,^(29)^ the 45-minute cognitive battery test, the Positive and Negative Affect Schedule-NOW (PANAS-NOW), and the computerised Mental Performance Assessment System were used to measure mood and cognitive functions.^(17)^

### Overview of the effect of dietary intake of fruits and vegetables on cognitive functions

#### Directionality of association

Within the examined literature different studies have been conducted evaluating the directionality of relationships between dietary intake of FAV and cognitive functions. Among the six included studies,^(17,28–32)^ one study viewed cognitive performance as the primary outcome,^(31)^ whereas the other two studies.^(28,32)^ FAVs within dietary patterns are a cause of some aspects of cognitive functions. Studies^(17,29,30)^ on the consumption of fruit juices have shown that it is effective in both memory and cognition

### Main findings on the specific association between dietary intake of FAV and cognitive functions

The included studies explicitly described the relationship between dietary intake of FAV on cognitive function among adolescents and adults. A detailed summary of the intake of fruits and vegetables from the included studies is presented in Table 2.

### Dietary patterns as exposure measures and cognitive functions as an outcome

Among the six studies,^(17,28–32)^ three included longitudinal, prospective, and cohort studies. A prospective cohort study by Nyadri et al.^(28)^ reported the potential links between dietary patterns and cognitive performance among adolescents. The study reported that the Western diet is known for consuming high amounts of fast food, red meats, and processed items. Regarding cognitive performance, the Western type of diet was negatively associated with cognitive performance, while eating more fruits and leafy green vegetables was positively associated with mental performance. Additionally, Individuals with high dietary intake of the Western diet at 14 years of age showed lower cognitive performance at a later age.

Chen Jing et al.^(30)^ conducted a cohort study that reported the dietary intake from 1980 to 2011 and assessed cognition in 2011 with six dietary patterns from 24 hours of diet recall, and it found that both youth and long-term vegetable and dairy product consumption was positively associated with episodic memory and associative learning scores (p < 0.05). Compared with vegetables and dairy products (p < 0.05), long-term high-carbohydrate patterns were negatively related to sustained attention and visual processing. In contrast, higher adherence to healthy vegetable and dairy product patterns was associated with better cognitive function. The findings suggest that maintaining a healthy diet from early life to adulthood promotes cognitive health.

### Fruits and vegetables as an exposure measure and cognitive function as an outcome

Mao Xuanxia^(31)^ conducted a cohort study on 3231 young adults aged 18–30 years to evaluate the long-term associations between cognitive function FAVs intake and fruit-vegetable subgroup intake. Diet was assessed at baseline, 7 years, and 20 years. At assessment year 25, cognition was evaluated using three cognitive tests. The study’s results showed that, except for eating potatoes, eating entire veggies was strongly associated with improved cognitive performance (p = 0.01); furthermore, fruits, except for fruit juices, were significantly associated with improved cognitive performance (p = 0.03). Aside from potatoes and fruit juice, which have relatively little fibre, most VFs are high in fibre.

### Fruit juices as exposure measures and cognitive functions as an outcome

Of the six studies conducted,^(17,28–32)^ three^(17,29,30)^ reported on the improvement of cognition about the consumption of fruit juices. Daniel Lamport J. et al. reported that twenty-four young adults, aged 18–30, who were in good health, were seen to score significantly better on the digit symbol substitution test two hours after consuming the high flavanone drink as compared to the baseline and control beverage. Nevertheless, no differences were seen in any other behavioural or cognitive test results. These findings show that in young, healthy adults, consuming flavanone-rich citrus juice in amounts that are typically consumed can significantly improve blood flow to the brain.

Even purple grapes are effective at improving cognitive function and mood in healthy adults. Ramsay Haskell C. F. reported that compared with placebo, purple grape juice significantly improved some cognitive functions, such as reaction time, according to a composite attention measure (p = 0.047) and increased calmness ratings (p = 0.046). Positive effects on pre-dose memory reaction time (p = 0.018) and post-dose calm ratings (p = 0.019) were found when purple grapes were consumed first. Thus, the findings in healthy young adults suggest that purple grape juice can acutely enhance cognition and mood.

Khalid Sundus et al. reported that flavonoids (which are typically found in FAVs) are associated with a reduced risk of depression. One possible explanation for this relationship is that flavonoids have been shown to improve executive function (EF), which is linked to depression-related cognitive processes that sustain depression and low mood. Improved EF may reduce the depression process and thus improve mood. This study demonstrated the acute effects of blueberry flavonoid consumption on positive affect and no effect negative affect in healthy children and young adult.

However, the association between dietary intake of FAV remains inconclusive. Many studies have shown that decreased dietary intake of FAV leads to decreased cognitive impairment,^(33)^ However, very few studies have been performed on adolescents and young adults. In this longitudinal study^(31)^ on direct intake of FAV, the consumption of entire vegetables was associated with improved cognitive performance, which was measured through a standardised test with a follow-up, a baseline, and seven and 25 years. However, in other studies, only specific food groups, such as those related to the consumption of mushrooms, were associated with some domains of cognitive function, such as reasoning tests and memory^(34)^; one additional study emphasised the intake of green leafy vegetables, and more fruits were positively associated with some aspects of cognitive performance.^(28)^

Educating adolescents on consuming FAVs early is important to prevent cognitive decline during midlife. Cognitive decline, an ageing-related condition, is a risk factor for dementia. Because of their varying nutrient content, different fruits and vegetables have diverse effects on cognitive functioning.^(35)^ The authors found that only limited articles and data focused on the dietary intake of FAV among adolescents and adults. This also indicates that future studies should be conducted in different contexts and cultures. There is a need to increase the daily intake of FAV among adolescents at a very early age through modifiable risk factors such as improved dietary habits to prevent early cognitive decline.

## Discussion

To the best of our knowledge, this scoping review is the first attempt to determine the intake of FAV intake on cognitive performance in adolescents and adults. The strengths of the present review include the thorough search of both published and unpublished literature, as well as the strict methodology for screening and the inclusion of every potential study over the past 10 years. In most studies, the link between FAV and cognitive functions was not the focus of the analysis; instead, a cohort design and a longitudinal study design were used. Despite these drawbacks, the literature highlights how FAV and cognitive functions are related. FAVs are essential for our daily diet, and a lack of proper diet has considerable negative consequences on cognitive and mental health at the individual and population levels; thus, according to the available data, although generally good for cognitive functioning, FAV may have varied effects on different cognitive domains over the course of a person’s life. These different benefits need to be further studied. Hence, additional research in this area is needed.

A quantitative evaluation of the connection between FAV and cognitive functions has been conducted, but very few studies have evaluated this topic in adolescents and young adults. FAVs are rich in various carotenoids, phenolic vitamins, and minerals required for all age groups; thus, consuming fruits and vegetables is highly beneficial.^(35,36)^ According to one study, the amount of fruit consumed by the mother during pregnancy is connected with a 2.38-point increase in cognitive growth over a year.^(37)^ Visual and spatial skills^(38)^ improve early childhood intelligence, and executive function increases,^(39)^ and developmental delay is prevented.^(40)^ Additionally, it benefited children and young people by improving their academic performance.^(9)^ A history of higher fruit consumption over a year was linked to greater academic achievement across all age groups. The intake of a high-quality diet is also very important for improving academic achievement in adolescence,^(10,41)^ but very few studies have investigated this topic. The consumption of antioxidant-rich foods has a vital impact on elderly people’s performance. This indicates that quality food consumption can be expected to enhance the quality of life of elderly people. The older population consumes adequate amounts of FAV to reduce oxidative stress, the main cause of cognitive impairment.^(42)^ Thus, the consumption of FAV is required in all age groups, but there is a paucity of research on the effect of consuming fruits and vegetables on cognitive development, especially among adolescents and young adults.

Multiple studies have assessed the intake of fruits and vegetables among adolescents and adults. Previous research has focused mainly on micronutrients,^(43)^ intake of breakfast, and consumption^(3,11)^ of fish in association with cognitive functions among adolescents^(34,44,45)^

Some studies have reported that the consumption of fast noodles and beverages was negatively correlated with several cognitive parameters.^(32,45,46)^ Thus, the consumption of mushrooms has been found to have a positive effect on cognitive tests. According to related research, there is a stronger correlation between food intake and cognitive function in women than in men.^(34)^ Thus, maintaining healthy cognitive abilities is associated with eating well-balanced meals. Adolescents eat junk food for three days or more a week, and they are more attracted to junk foods such as biscuits, noodles, cookies, chips, cakes, ice cream, chocolate chow, mien, samosa, coke, Pepsi, burgers, pizza, canned foods, meat products and fried potatoes.^(47,48)^ Currently, adults are attracted to more Western-pattern types of diets, as in this review, a prospective study was performed in which increased consumption of Western foods at an early age was also associated with diminished cognitive function as age advanced. French fries, potato chips, and fried potatoes exert a significant influence on cognitive performance, while FAV has a positive influence.^(28)^In light of this, the same study’s results suggest that, depending on the adult’s diet intake, there may be a connection between dietary practices and some aspects of cognitive performance^(49,50)^.

The intake of whole vegetables such as deep yellow, dark green vegetables, tomatoes, avocado, and guacamole is associated with better cognition. The study also examined fibre intake and cognitive function. Phytochemicals found in dietary fibres may halt the onset and progression of neurodegenerative illness, as many others, possibly leading to slow cognitive loss, as indicated by glucose concentration, blood pressure, insulin sensitivity, and serum lipids. Dietary fibre consumption^(51)^ has been linked to several positive health impacts, including a decreased risk of obesity and CVD.^(52)^ Most published studies have not evaluated VF subgroups based on dietary fibre intake, which may account for, at least in part, the contradictory findings in the literature. There is controversy regarding the intake of fruit and fruit juice. This study showed that the intake of fruits, except for fruit juice, was significantly associated with better cognitive function. Few studies have investigated fruit juices such as berries,^(29)^ purple grapes,^(17)^ and flavonoids^(30)^ to promote cognition. A thorough evaluation also revealed that drinking fruit juice provided equivalent protection against cognitive deterioration as eating whole fruits^(30)^.

According to a continuous scale spanning from normal aging to dementia, individuals with poor cognitive performance in midlife have an elevated risk of dementia later in life.^(53)^ The peer group had a greater influence on adolescents’ lifestyle and nutritional choices and Western patterns of diet, such as soft drinks, red and processed meat, which are high in saturated and total fat refined sodium, and sugar, as explored in our prospective study,^(28)^ Thus, tracking a cohort of young adults between 10 and 30 years of age provides a once-in-a-lifetime opportunity to investigate the early cause of cognitive decline. Chronic cognitive decline occurs with age, and long-term randomised trials may be impractical. The longitudinal and population-based cohort studies^(17,29,30)^ included in this review provide vital data to better understand cognition in association with diet. Additionally, very few studies have examined VFs separately across separate cognitive domains. In the current analysis, one study revealed separate outcomes for VAF high and low in dietary fibre, as well as for vegetables rich in various antioxidants. These findings provide robust human data supporting the cognitive benefits of dietary fibre as well as the favourable impacts of lycopene and carotenoids. Additionally, the study revealed a positive relationship between vegetable consumption and several aspects of cognitive function. The review also emphasised the benefits of the intake of FAV and the negative effects of unhealthy foods on cognitive functions.

### Literature review gaps and recommendations

Several gaps were identified in the articles that were reviewed. First, a few articles were found in the literature review. Three studies were randomised controlled trials.^(17,29,30)^ Three were longitudinal studies,^(28,31,32)^ which made it difficult to determine a connection between FAV and cognitive functions. To better comprehend the potential connections between FAV and cognitive function problems in adolescents and young adults, additional interventions, especially more longitudinal studies, are needed. Second, it is necessary to investigate the relationship between FAV and particular cognitive domains (memory, learning, attention, decision-making, and language abilities). FAV might not have the same effect on each cognitive domain.^(24)^

Previous research on the adult population revealed a positive correlation between verbal memory scores and FV intake, fruit intake, vitamin C &E intake, and vitamin C-rich FVs,; In contrast, executive functioning scores were negatively correlated with intakes of FV, vegetable intake alone, and FV’s high in b-carotene^(54)^ These findings have thus far been found to be disproportionately prevalent in studies of adults.

Third, the countries where the relevant studies were published were the UK,^(17,29,30)^ the USA,^(31)^ Australia^(28)^ and Finland^(32)^; consequently, we have little knowledge of how the dietary intake of FAV differs in various socioeconomic, cultural, and political contexts according to the dietary recommendations of each country. This highlights the need for further study in this area in other low- and middle-income countries and geographical regions where there may be an intolerably high prevalence of nutritional deficiencies, malnutrition, and cognitive impairments, particularly at particular rates.

Fourth, one study focused on specific healthy and Western dietary patterns,^(28)^ except for the studies included in this review that focused on FAV. Thus, another study overrepresented the dietary intake subgroup,^(31)^ three studies^(17,29,30)^ focused on the consumption of fruit juices, and only two longitudinal studies reported that the intake of fruits and vegetables through adulthood is associated with improved cognitive function in midlife.^(31,32)^ Therefore, additional data are not available to specify that there is a specific link between dietary intake and cognitive function.

Fifth, studies examining these specific associations are necessary because the majority of earlier studies did not pay much attention to the connection between FAV and CF. Thus, conducting primary research with more interventional or longitudinal studies is important.

Sixth, additional efforts must be made to identify the specific food groups that enhance cognitive function and the specific cognitive domains and thus the relationship between FAV and CF however, additional interventional and cohort studies are needed.

Finally, the findings of the present scoping review suggest a link between FAV and cognitive functions among adolescents and young adults. Therefore, it is essential to promote and support access to nutritional counselling and health education on nutrition and its effects on cognitive functions in all age groups to prevent further cognitive impairment in later years of life. The following limitations apply to the current scoping review. First, only peer-reviewed English-language papers were included. Second, children included at baseline and young adolescents older than 35 years were examined in this review, and neither adults older than 35 years nor elderly individuals were considered. Due to our desire for more comprehensive systematic reviews, we did not assess the methodological quality of the included studies. Finally, due to the small number of studies, i.e., randomised controlled trails and longitudinal designs, and the limited contextual settings of the studies included in this review, the results may not be generalisable.

In conclusion, the findings of this scoping review suggest a potential association between the dietary intake of FAV and cognitive function among adolescents and young adults. More longitudinal, intervention, and cohort-based studies are needed to understand better the nature and direction of the relationships between FAV and cognitive functions in this population. There is a need for additional research among different populations and countries. This study has implications for nutrition policy, as it draws attention to strengthening the nutritional status and nutritional habits of adolescents and young adults, which remain significant issues in most developing countries.

## Supporting information

Soans et al. supplementary material Soans et al. supplementary material

## Supplementary material

The supplementary material for this article can be found at https://doi.org/10.1017/jns.2025.10012
