# Supplementary material for: Intake of fruits and vegetables (FAVs) on cognitive functions among adolescents and young adults: a scoping review
Source: J Nutr Sci. 2025 Nov 20;14:e82. doi: 10.1017/jns.2025.10012 (PMC12658299; doi:10.1017/jns.2025.10012)
Supplement: Soans et al. supplementary material [file S2048679025100128sup001.docx]

**Appendix File: Dietary intake of fruits and vegetables and cognitive functions Outcomes among Adolescents and adults: A Scoping Review.**

**Appendix A: Search terms used for the literature search.**

| Population | "Adolescent" OR Adolescence* OR “Adult Children" OR “Young Adult" OR Teen*OR Youth*OR “Young person” OR Youngsters |
| --- | --- |
| Intervention | "Eating “OR "Feeding Behavior*" OR “dietary intake*” OR "Fruit" OR "Vegetables" |
| Outcome | Cognition OR "Cognitive Function" OR Mood* OR "Perception" OR "Attention" OR "Memory" OR "Decision Making" OR "Language" OR "Executive Function" OR Cognition |

**Appendix B. Search terms used for each database.**

| **Database** | **Search strategy** | **Search period** | **Limits** |
| --- | --- | --- | --- |
| PubMed | "Eating"[Mesh] OR "Dietary intake “OR "Food intake “AND "Fruit"[Mesh] OR Fruit* OR "fruits, vegetables" AND Vegetable*OR "Vegetables"[Mesh] AND "Cognition"[Mesh] OR "Cognitive Function" OR Mood* OR "Perception"[Mesh] OR "Attention"[Mesh] OR "Memory"[Mesh] OR "Decision Making"[Mesh] OR "Language"[Mesh] AND "Adolescent"[Mesh] OR Adolescen* OR "Adult Children"[Mesh] OR "Young Adult"[Mesh] OR Teen*OR Youth* OR "Young person" OR Youngsters | 2012/01/01 to 2024/12/30 | Humans, English |
| CINHAL | MM "Eating" OR "Eating"  OR MM "Food Intake+" OR "Food intake" OR "Dietary intake" AND MM "Fruit+" OR "Fruit" OR MM "Fruit Juices+" OR MM "Vegetables+" OR "Vegetables" OR fruits and vegetables AND MM "Cognition+" OR "Cognitive Function" OR MM "Decision Making+" OR MM "Attention+" OR MM "Perception+") OR MM "Memory+" OR MM "Language “OR "Mood" MM "Adolescence+" OR MM "Childbearing Age" OR (MH "Child") OR (MM "Minors (Legal)") OR (MM "Young Adult") (MM "Adult Children") OR teen OR teens OR adolescents OR teenagers | 2014/01/1 to  24/2/28 |  |
| Scopus | ( ALL ( "Eating"  OR  "Dietary intake"  OR  "Food intake" ) )  AND  ( ALL ( "Fruit"  OR  fruit* ) )  AND  ( ALL ( "Vegetables"  OR  vegetable* ) )  AND  ( ALL ( "Cognition"  OR  "Cognitive Function"  OR  mood*  OR  "Perception"  OR  "Attention"  OR  "Memory"  OR  "Decision Making"  OR  "Language" ) )  AND  ( ALL ( "Adolescent"  OR  adolescen*  OR  "Adult Children"  OR  "Young Adult"  OR  teen*  OR  youth*or  "Young person"  OR  youngsters ) )  AND  ( LIMIT-TO ( PUBYEAR ,  2024 )  OR  LIMIT-TO ( PUBYEAR ,  2022 )  OR  LIMIT-TO ( PUBYEAR ,  2021 )  OR  LIMIT-TO ( PUBYEAR ,  2019 )  OR  LIMIT-TO ( PUBYEAR ,  2018 )  OR  LIMIT-TO ( PUBYEAR ,  2017 )  OR  LIMIT-TO ( PUBYEAR ,  2016 )  OR  LIMIT-TO ( PUBYEAR ,  2015 )  OR  LIMIT-TO ( PUBYEAR ,  2014 )  OR  AND  ( LIMIT-TO ( EXACTKEYWORD ,  "Human" ) )  AND  ( LIMIT-TO ( LANGUAGE ,  "English" ) ) | 2014/02/1 to  24/2/28 |  |
| Web of science | **ALL= (Adolescent OR adolescence OR teen OR teens OR teenagers OR teenage OR youths OR youth OR young OR women OR youngsters OR female OR "youthful Adolescents Female" OR "Adolescent Female" OR "Female Adolescent" OR "Female adolescents" OR youthful) AND ALL= (Eating OR "Feeding Behavior" OR "dietary intake" OR Fruit OR Vegetables OR Fruit OR Vegetables) AND ALL=(Cognition OR "Cognitive Function" OR "Decision Making" OR Attention OR "Perception OR Memory OR Language")** or **2024** or **2023** or **2022** or **2021** or **2020** or **2019** or **2018** or **2017** or **2016** or **2015** or **2014** (Publication Years) and **Article** (Document Types) and **English** (Languages) | 2014/02/1 to  24/2/28 |  |
| EMBASE | ‘Eating’/exp OR ‘Dietary intake/exp OR "Food intake” /exp AND "Fruit"/exp  OR Fruit OR "fruits, vegetables"/exp AND Vegetable/exp *AND "Cognition"/exp OR "Cognitive Function" OR Mood/exp OR "Perception"/exp OR "Attention"/exp OR "Memory"/exp OR "Decision Making"/exp OR "Language"/exp AND "Adolescent”/exp OR “Adolescence”/exp OR "Adult Child”/exp OR Teen OR Youth OR ‘Young person’ youngsters AND 31 AND (2014:py OR 2015:py OR 2016:py OR 2017:py OR 2018:py OR 2019:py OR 2020:py OR 2021:py OR 2022:py R 2023:py OR 2024:py o) AND [humans]/lim AND [english]/lim | 2014/02/1 to  24/2/28 |  |
